# Crosstalk between diacylglycerol kinase and protein kinase A in the regulation of airway smooth muscle cell proliferation

**DOI:** 10.1186/s12931-023-02465-8

**Published:** 2023-06-10

**Authors:** Miguel A. Hernandez-Lara, Santosh Kumar Yadav, Stanley Conaway, Sushrut D. Shah, Raymond B. Penn, Deepak A. Deshpande

**Affiliations:** Department of Medicine, Center for Translational Medicine, Jane & Leonard Korman Respiratory Institute, Sidney Kimmel Medical College, Thomas Jefferson University, Philadelphia, PA 19107 USA

**Keywords:** Diacylglycerol kinase, Protein kinase A, Airway smooth muscle, Airway remodeling, Proliferation

## Abstract

**Background:**

Diacylglycerol kinase (DGK) regulates intracellular signaling and functions by converting diacylglycerol (DAG) into phosphatidic acid. We previously demonstrated that DGK inhibition attenuates airway smooth muscle (ASM) cell proliferation, however, the mechanisms mediating this effect are not well established. Given the capacity of protein kinase A (PKA) to effect inhibition of ASM cells growth in response to mitogens, we employed multiple molecular and pharmacological approaches to examine the putative role of PKA in the inhibition of mitogen-induced ASM cell proliferation by the small molecular DGK inhibitor I (DGK I).

**Methods:**

We assayed cell proliferation using CyQUANT™ NF assay, protein expression and phosphorylation using immunoblotting, and prostaglandin E_2_ (PGE_2_) secretion by ELISA. ASM cells stably expressing GFP or PKI-GFP (PKA inhibitory peptide-GFP chimera) were stimulated with platelet-derived growth factor (PDGF), or PDGF + DGK I, and cell proliferation was assessed.

**Results:**

DGK inhibition reduced ASM cell proliferation in cells expressing GFP, but not in cells expressing PKI-GFP. DGK inhibition increased cyclooxygenase II (COXII) expression and PGE_2_ secretion over time to promote PKA activation as demonstrated by increased phosphorylation of (PKA substrates) VASP and CREB. COXII expression and PKA activation were significantly decreased in cells pre-treated with pan-PKC (Bis I), MEK (U0126), or ERK2 (Vx11e) inhibitors suggesting a role for PKC and ERK in the COXII-PGE_2_-mediated activation of PKA signaling by DGK inhibition.

**Conclusions:**

Our study provides insight into the molecular pathway (DAG-PKC/ERK-COXII-PGE_2_-PKA) regulated by DGK in ASM cells and identifies DGK as a potential therapeutic target for mitigating ASM cell proliferation that contributes to airway remodeling in asthma.

## Introduction

Airway remodeling is a major component of asthma pathogenesis that includes structural changes such as increased airway wall thickening [1]. Increased airway smooth muscle (ASM) mass caused by ASM hyperplasia and hypertrophy contributes significantly to airway wall thickening and causes increased resistance to airflow [1]. Thus, therapeutically targeting ASM remodeling has emerged as a promising approach for severe asthma treatment [2]. Identifying novel regulators of ASM proliferation is critical in developing anti-remodeling therapies for use in asthma.

ASM cell proliferation is regulated by multiple airway inflammatory mediators including growth factors, cytokines, chemokines, and other proteins and peptides that are agonists of G protein-coupled receptors (GPCR) and growth factor receptors that induce promitogenic signaling in ASM cells [3–12]. These mediators include Gq-coupled GPCR agonists such as thrombin, leukotrienes, and growth factors such as platelet-derived growth factor, epithelial growth factor, and vascular growth factor [3–5, 13]. Growth factors promote ASM cell growth via activation of receptor tyrosine kinase (RTK) pathways whereas GPCR agonists promote Gq-mediated activation of phospholipase C [14]. A previous study from our lab demonstrated synergy between RTK and Gq-coupled GPCR signaling pathways in regulating ASM cell proliferation [3–5]. Activation of Gq signaling (as well as RTK signaling) in ASM cells results in the activation of phospholipase C which in turn converts phosphoinositide bis phosphate into inositide tris-phosphate and diacylglycerol (DAG) [14]. DAG-mediated signaling is regulated by a class of lipid kinase enzymes belonging to DAG kinase (DGK) [15, 16]. Recent studies from our lab have demonstrated that α and ζ isoforms of DGK play a central role in the regulation of ASM cell contraction [17, 18], proliferation [19], and allergen-induced airway inflammation, hyperresponsiveness, and features of airway remodeling in mice [19, 20] using molecular (knockout and knockdown) and pharmacological (DGK inhibitor) approaches. We demonstrated that DGK inhibition perturbs the stoichiometry of phospholipids DAG and phosphatidic acid (PA) in ASM cells and regulates ASM cell proliferation [19]. Interestingly, our studies demonstrated an anti-mitogenic effect of DGK inhibition despite promoting DAG-mediated activation of protein kinase C (PKC) and extracellular signal-regulated kinase (ERK)-mediated signaling, which is typically pro-mitogenic signal [19]. Therefore, in this study, we aimed to delineate molecular pathways that are involved in the inhibition of ASM cell proliferation by DGK.

Previous studies have shown that agonists of Gs-coupled GPCRs inhibit ASM cell proliferation and protein kinase A (PKA) plays a central role in this process [21–24]. Activation by Gs-coupled GPCR by various agonists such as beta-agonists, prostaglandin E_2_ (PGE_2_) results in the activation of heterotrimeric Gs and dissociation of its α and βγ subunits [21–25]. The α subunit directly interacts with and activates adenylyl cyclase, which hydrolyzes ATP to generate cyclic adenosine monophosphate (cAMP). cAMP inhibits ASM cell proliferation via activation of the cAMP-dependent protein kinase, PKA [24]. Thus Gs-cAMP-PKA is a predominant anti-mitogenic pathway in ASM cells. However, the role of DGK inhibition-induced Gs signaling in ASM cell proliferation is not known. Therefore, herein we sought to investigate the role of cAMP-PKA in DGK-mediated regulation of ASM cell proliferation. In our published paper, in the context of contractile inhibition by acute DGK inhibition, we noted a cyclooxygenase II (COXII) induction and PKA activation and we therefore hypothesized that PKA mediates the anti-mitogenic effect. Our findings presented in this manuscript demonstrate that chronic pharmacological inhibition of DGK increase paracrine secretion of PGE_2_ via a PKC/ERK1/2-dependent induction of COXII. PGE_2_ in turn promotes cAMP-PKA signaling to inhibit ASM cell proliferation. These findings establish a novel mechanism by which temporal regulation of DGK cross-talks with Gs-PKA pathway to regulate ASM cell proliferation and further advances DGK as a potential therapeutic target to mitigate ASM remodeling associated with asthma.

## Materials and methods

### Materials

The primary antibody against VASP (610448) was from BD biosciences. Antibody for p-CREB (9198S) and COXII (12282S), human Platelet-Derived Growth Factor BB (PDGF-BB) (8912), and RIPA cell lysis buffer (9806) were purchased from Cell Signaling Technology (Beverly, MA, USA). Primary antibody against β-actin (58522) and diacylglycerol kinase Inhibitor I (R59022, referred as DGK I) were purchased from Sigma (St. Louis, MO, USA). Secondary antibodies IRDye 680RD or 800CW were from LI-COR (Lincoln, NE, USA). Insulin-transferrin-selenium (ITS) was from Thermo Fisher Scientific (41400045; Waltham, MA, USA). Protease and phosphatase inhibitors were from Bimake (Houston, TX, USA). All polyacrylamide gel casting, running, and transfer reagents and equipment were from Bio-Rad Laboratories (Hercules, CA, USA) or previously identified sources [22, 26]. CyQuant cell proliferation assay kit was from Life Technologies (Grand Island, NY, USA). PGE_2_ competitive ELISA (# 514010) was from Cayman Chemicals (Ann Arbor, MI, USA).

### Cell culture

Human ASM cells were isolated from de-identified lung donors and cultured (passage 2–6) using HAM’s F-12 media supplemented with 10% FBS, penicillin/streptomycin, HEPES buffer, CaCl_2_, l-Glutamine (Gibco), and NaOH. Human ASM cell cultures were replenished with fresh media every 2 days [27]. Cells were cultured until 80% confluency and serum-starved with HAM’s F-12 medium containing 1% ITS for 24 h before experiment.

### Viral transfection of human ASM cells

Retrovirus for the expression of green fluorescent protein (GFP) or the GFP-chimera of the PKA inhibitory peptide (PKI) was produced by co-transfecting GP2-293 cells with p-vesicular stomatitis virus (VSV)-G vector (encoding the pantropic VSV-G envelope protein) and either pLPCX-GFP or pLPCX-PKI-GFP. Culture media containing viral particles were harvested after 48 h of transfection and used to infect primary human ASM cell cultures as described previously [22, 23, 28]. Human ASM cell cultures were subsequently selected for homogeneity with puromycin (5 µg/ml) (Tocris Bioscience, UK).

### Cell proliferation assay

Human ASM cells were plated in a 96-well plate for CyQuant assay at a density of 5000 cells per well and maintained in complete Ham’s F-12 medium supplemented with 10% FBS. After 24 h, cells were serum-starved with HAM’s F-12 medium containing 1% ITS and treated with PDGF (10 ng/ml) or DGK I (0–20 µM) + PDGF (10 ng/ml) for 48 h. A subset of cells was also treated with DMSO (vehicle control).

### Western blotting

Human ASM cells were plated in 12-well plates and maintained in HAM’s F-12 + 10% FBS media for 24 h. Cells were then serum-starved with HAM’s F-12 medium containing 1% ITS for 24 h followed by pre-treatment with vehicle or pan-PKC (Bis I), MEK (U0126) or ERK2 (Vx11e) inhibitor for 10 min, and then stimulated with vehicle or DGK I (20 µM) for 24 h. In a subset of experiments, cells were stimulated with vehicle or DGK I (20 µM) for 0, 5, 10, 15 and 30 min, or 24 h. Human ASM cells were lysed in RIPA buffer (CST, USA) supplemented with protease and phosphatase inhibitor (Bimake) at 4 °C for 30 min. Lysates were then mixed with Laemmli buffer (Bio-Rad) containing 10% β-mercaptoethanol and boiled at 95 °C for 5 min. Samples were separated on SDS-PAGE, and transferred onto a nitrocellulose membrane and blocked using 3% BSA in TBST for 1 h. Target proteins were detected via incubation with antigen-specific primary antibodies [COXII (1:1000), p-CREB (1:1000), VASP (1:5000), p-ERK1/2 (1:1000), β-actin (1:50,000)] overnight in 3% BSA in TBST. A secondary antibody (1:10,000 in 3% BSA in TBST) conjugated with an infrared dye, either at 680 or 800 nm wavelength, was used to detect target protein using Odyssey infrared scanner (LI-COR Biosciences, Lincoln, USA). Protein band intensity was quantified using Odyssey software as described previously [19].

### PGE_2_ assay

Human ASM cells were seeded in 12-well plates, and serum-starved with HAM’s F-12 medium containing 1% ITS 24 h later. Cells were treated with vehicle or DGK I (20 µM) for 30 min–24 h. The supernatants were collected at different time points and assayed for PGE_2_ content using a competitive ELISA approach (Ann Arbor, MI, USA) according to the manufacturer’s protocol.

### Statistical analysis

All data are presented as mean ± SEM values from ‘n’ number of lines derived from distinct lung donors. Individual data points from a single experiment were calculated as the mean value from 3 replicate observations for CyQuant assay and data were reported as fold change from the basal conditions. Densitometry data of western blot are normalized using band intensities to vehicle-treated cells. PGE_2_ concentrations were determined by extrapolation from a standard curve. One-way ANOVA with Bonferroni post-hoc analysis was used to determine statistical differences among treatment groups using GraphPad Prism IX software (La Jolla, CA, USA). A p ≤ 0.05 was considered sufficient to reject the null hypothesis.

## Results

### DGK inhibition attenuates proliferation in GFP-, not PKI-GFP-, expressing ASM cells

**Fig. 1:**
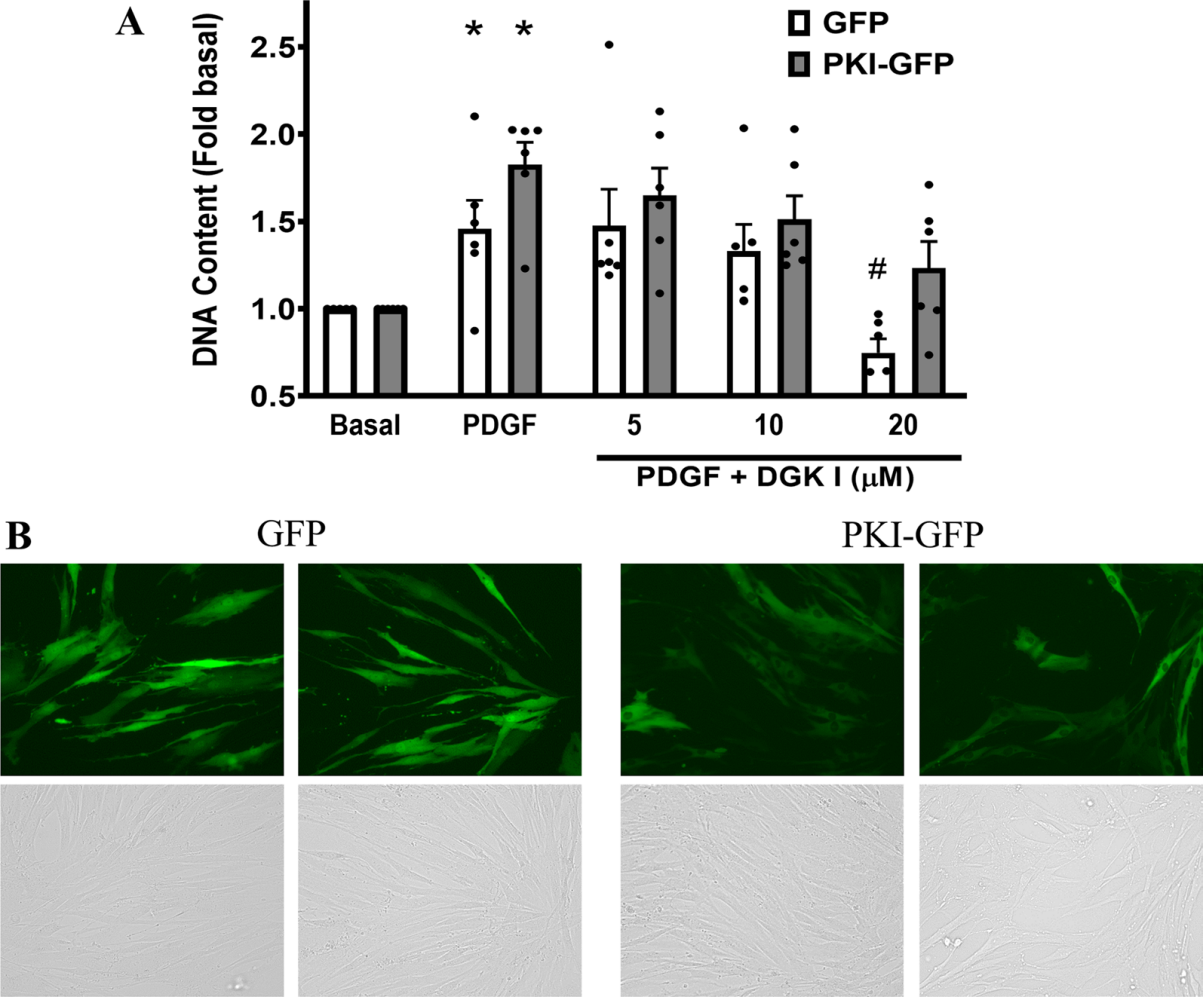
DGK inhibitor attenuation of ASM cell proliferation involves PKA. GFP- and PKI-GFP-expressing human ASM cells were pretreated with DGK I (0–20 µM) followed by PDGF (10 ng/ml) stimulation for 48 h and cell proliferation was measured by assessing total DNA. Graphical representation is of mean ± SEM (n = 6 distinct donors) of CyQuant fluorescence normalized to basal (**A**). Expression of GFP in ASM cells transduced with retroviral particles was confirmed by fluorescence microscopy. Representative florescence (top) and brightfield (bottom) images of two different lines are shown (**B**). *p < 0.05 (compared to matched basal) and ^#^p < 0.05 (compared to matched PDGF) using one-way-ANOVA with Bonferroni *post-hoc* analysis

To examine the role of PKA in regulating ASM cell proliferation we utilized GFP or PKI-GFP chimera expressing cells. We previously established that expression of PKI peptide in ASM cells inhibits PKA activity efficiently [22, 24]. ASM cells were pretreated for 10 min with DGK I (5–20 µM) followed by stimulation with platelet-derived growth factor (PDGF) (10 ng/mL) for 48 h. DGK I inhibited PDGF-mediated ASM cell proliferation in GFP-expressing cells and not in PKI-GFP-expressing ASM cells (Fig. 1A). Transduction of GFP in human ASM cells was further confirmed by fluorescence microscopy (Fig. 1B). These data demonstrate that DGK inhibition attenuates PDGF-induced ASM cell proliferation, and PKA plays a critical role in the anti-mitogenic effect of DGK inhibition.

### DGK inhibition enhances cyclooxygenase II expression and PGE_2_ production in ASM cells

**Fig. 2:**
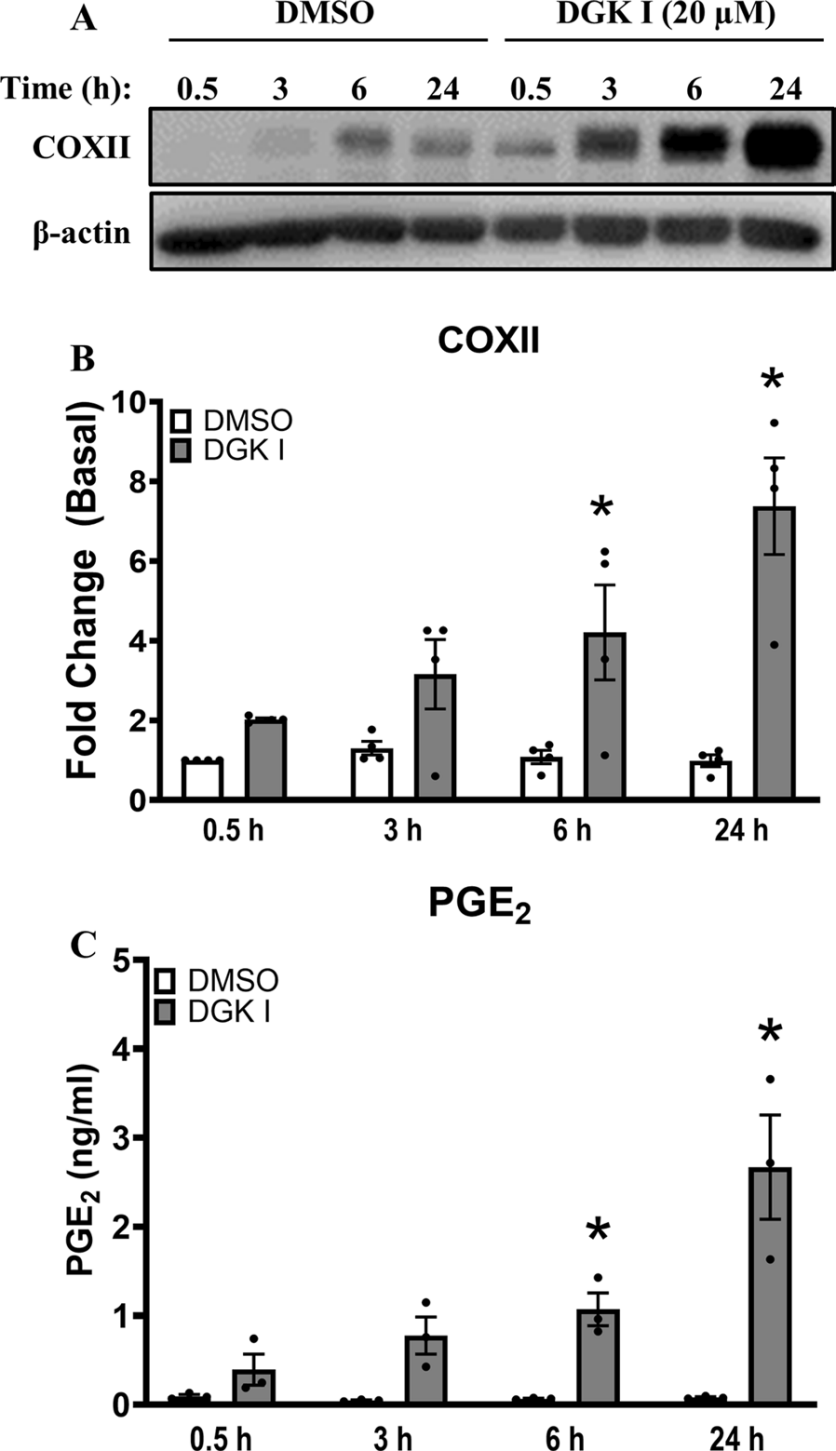
DGK inhibition enhances COXII expression and PGE_2_ production. Human ASM cells were treated with DGK I (20 µM) in a time-dependent manner. Cells were lysed and immunoblotted for COXII and β-actin (**A**). Graphical representation of mean ± SEM of COXII densitometry data normalized to basal conditions (**B**). Supernatant collected was subjected to ELISA to assay amounts of PGE_2_ (pg/ml) secreted and data were normalized to vehicle-treated condition (**C**). Data above are mean ± SEM (n = 3–4 distinct donors). *p < 0.05 (DMSO vs. DGK I at matched time points) using one-way-ANOVA with Bonferroni *post-hoc* analysis. Please note that the gel images shown are cropped from the full length images

**Fig. 3:**
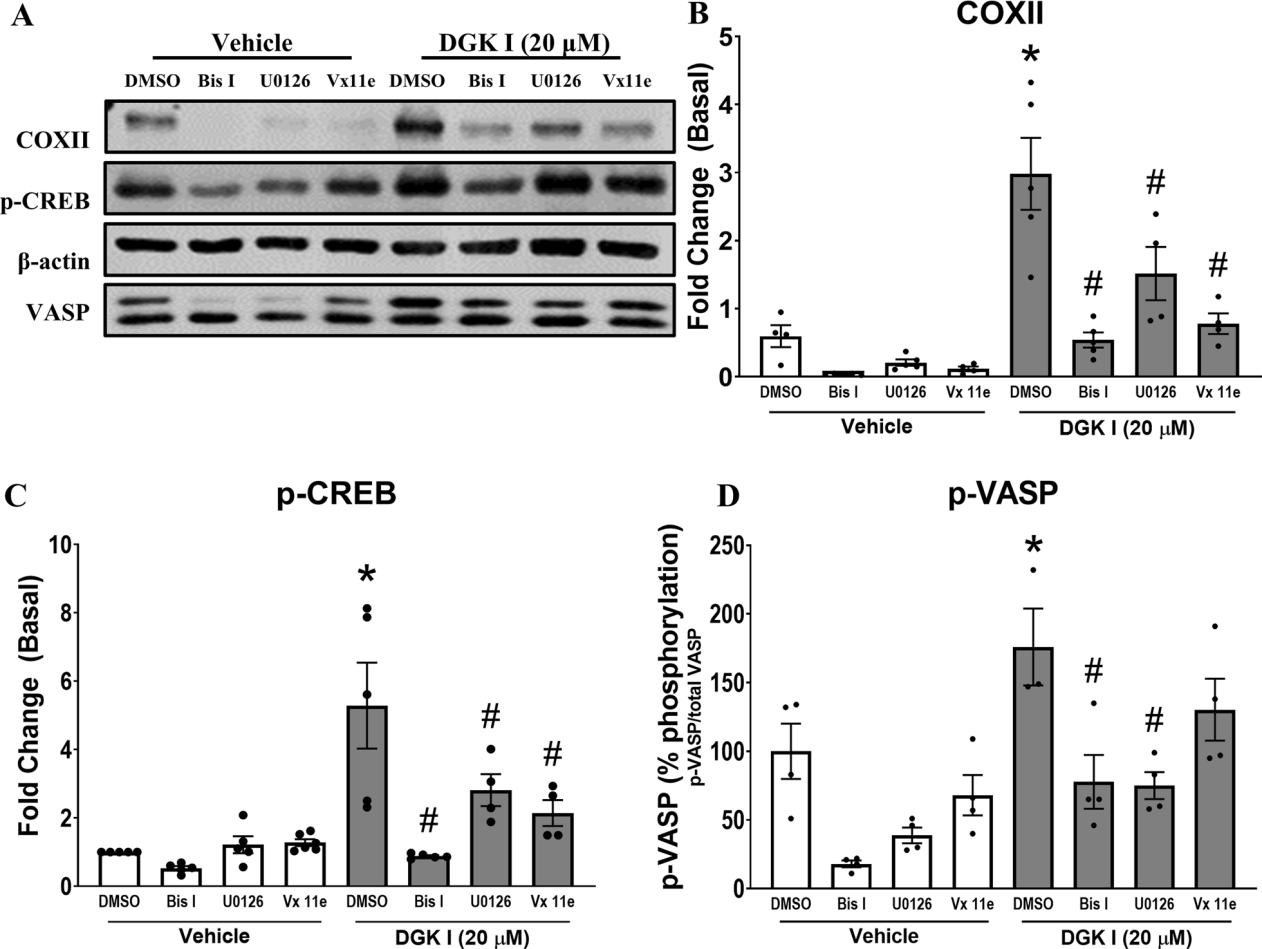
DGK inhibitor-induced COXII production and PKA activation is mediated via PKC-ERK1/2 signaling. Human ASM cells were pretreated with vehicle, Bis I (10 µM), U0126 (1 µM), or Vx11e (1 µM) for 10 min followed by stimulation with vehicle or DGK I (20 µM) for 24 h. Cells were lysed and immunoblotted for COXII, p-CREB, VASP, and β-actin (**A**). Graphical representation of mean ± SEM (n = 3–5 distinct donors) for COXII (**B**), p-CREB (**C**) and VASP (**D**). The densitometry data for COXII and p-CREB are normalized to basal conditions. Densitometry data for VASP phosphorylation are presented as percent (%) VASP shift (p-VASP/total VASP). *p < 0.05 (compared to vehicle basal) and ^#^p < 0.05 (compared to DGK I basal) using one-way-ANOVA with Bonferroni *post-hoc* analysis. Please note that the gel images shown are cropped from the full length images

Next, we aimed to establish a mechanism by which DGK inhibition mitigates ASM cell proliferation in a PKA-dependent manner. Inhibition of DGK results in accumulation of DAG species which are known to translocate protein kinase C (PKC) to the plasma membrane [18, 29, 30]. Membrane-bound PKC activates downstream effectors, including ERK1/2 in a Ras-Raf-MEK mechanism [14, 31]. Although ERK1/2 activation can promote cell proliferation, previous studies have shown a role for ERK1/2 in inducing expression of COXII which induces generation of PGE_2_, an agonist of Gs-coupled GPCRs EP2 and EP4 capable of PKA activation [17, 22, 24, 32–36]. Human ASM cells were treated with DGK I (20 µM) for different time periods ranging from 30 min to 24 h. Cell culture media was collected and cells were lysed to harvest total proteins. DGK I induced COXII expression in ASM cells in a time-dependent manner (Fig. 2A, B) compared to vehicle-treated ASM cells. Further, COXII induction was associated with an increase in PGE_2_ production from ASM cells treated with DGK I, but not in vehicle-treated cultures (Fig. 2C). Further, we assessed PKA activation by DGK I in ASM cells by immunoblotting for the phosphorylation of two key targets of PKA, vasodilator-stimulated phosphoprotein (VASP) and cAMP response element-binding protein (CREB). DGK inhibition significantly increased phosphorylation of CREB and of VASP (Fig. 3). Our data suggest that DGK inhibition results in COXII-mediated PGE_2_ production and activation of PKA signaling in ASM cells.

### DGK inhibition-induced COXII expression and PKA signaling are mediated by PKC activation

To gain further insight into the mechanism by which DGK inhibition induces COXII expression, we assessed the role of PKC. We hypothesized that DGK I-induced COXII induction, and subsequent activation of PGE_2_-Gs-PKA signaling involves activation of PKC based on the fact that DAG binds and activates PKC very efficiently, and DGK inhibition is expected to increase cellular DAG levels. Human ASM cells were pretreated with Bis I (10 µM), a pan-PKC inhibitor, for 10 min followed by treatment with vehicle or DGK I (20 µM) for 24 h. Cells were lysed and protein lysates were used for western blot analysis. There was a significant increase of COXII expression in the presence of DGK I which was significantly reduced in the presence of Bis I (Fig. 3A, B). To further test activation of PKA we additionally analyzed downstream effectors of PKA. There was a significant decrease in phosphorylation of CREB in the presence of Bis I in DGK I-treated ASM cells (Fig. 3A, C), as well as a significant reduction in DGK I-induced VASP phosphorylation (Fig. 3A, D). These data demonstrate DGK I-induced DAG activates PKC, leading to the increased expression of COXII and activation of PKA signaling.

### DGK inhibition-induced COXII expression and PKA signaling involves ERK-MAP kinase

Previous studies demonstrate that DAG-activated PKC induces activation of ERK1/2 by phosphorylation [37]. We hypothesized that DGK I-induced ERK1/2 activation mediates COXII induction and PKA activation. Human ASM cells were pretreated with vehicle, MEK1/2 inhibitor U0126 (1 µM), or ERK2 inhibitor Vx11e (1 µM) for 10 min followed by treatment with vehicle or DGK I (20 µM) for 24 h. Cells were lysed and protein lysates were used for western blotting. Our data demonstrate that DGK I alone induced COXII expression that was significantly inhibited in U0126 and Vx11e-treated cells (Fig. 3A, B). Moreover, induction of p-CREB and p-VASP by DGK I was significantly reduced when cells were pretreated with U0126, while Vx11e significantly reduced p-CREB and modestly reduced p-VASP (Fig. 3A, C, D). These data demonstrate DGK I-induced DAG activates ERK1/2, leading to the increased expression of COXII and activation of PKA signaling via paracrine production of PGE_2_.

### DGK inhibition increases ERK1/2 phosphorylation in a time-dependent manner

**Fig. 4:**
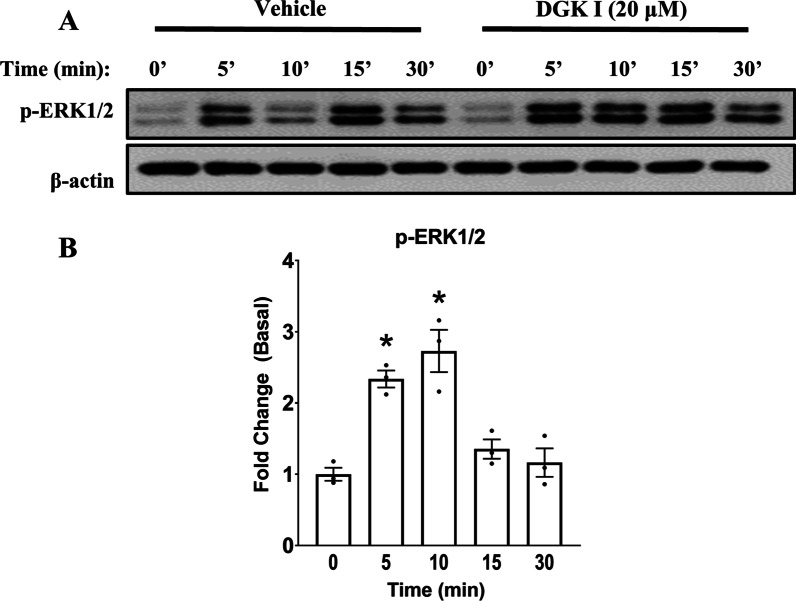
DGK inhibition activates ERK1/2 signaling in a time-dependent manner. Human ASM cells were treated with vehicle or DGK I (20 µM) for 0, 5, 10, 15 and 30 min. Cells were lysed and immunoblotted for ERK1/2 phosphorylation and β-actin (**A**). Graphical representation of mean ± SEM (n = 3 distinct donors) for ERK1/2 phosphorylation (**B**). The densitometry data for ERK1/2 phosphorylation by DGK I were normalized to matched vehicle-treated controls at each time point, and graphed as fold change from the basal. *p < 0.05 (compared to vehicle basal) using one-way-ANOVA with Bonferroni *post-hoc* analysis. Please note that the gel images shown are cropped from the full length images

To gain further insight on the role of ERK1/2 in DGK I- mediated COXII induction, we assessed activation of ERK1/2 by DGK inhibition. We hypothesized that treatment of cells with DGK I activates ERK1/2. Human ASM cells were treated with vehicle or DGK I (20 µM) for 0, 5, 10, 15, and 30 min. Cells were lysed and protein lysates were used for western blotting. Our data reveal that DGK I induced transient ERK1/2 phosphorylation and was significantly increased between 5 and 10 min (Fig. 4A, B) compared to vehicle and reached a basal level by 30 min. These data suggest that DGK inhibition indeed promotes ERK1/2 activity in a time-dependent manner. Collectively, our data demonstrate that DGK inhibition induces ERK1/2 activation leading to COXII induction, PGE_2_ secretion and PKA activation in human ASM cells. PKA activation results in inhibition of ASM cell proliferation.

## Discussion

**Fig. 5:**
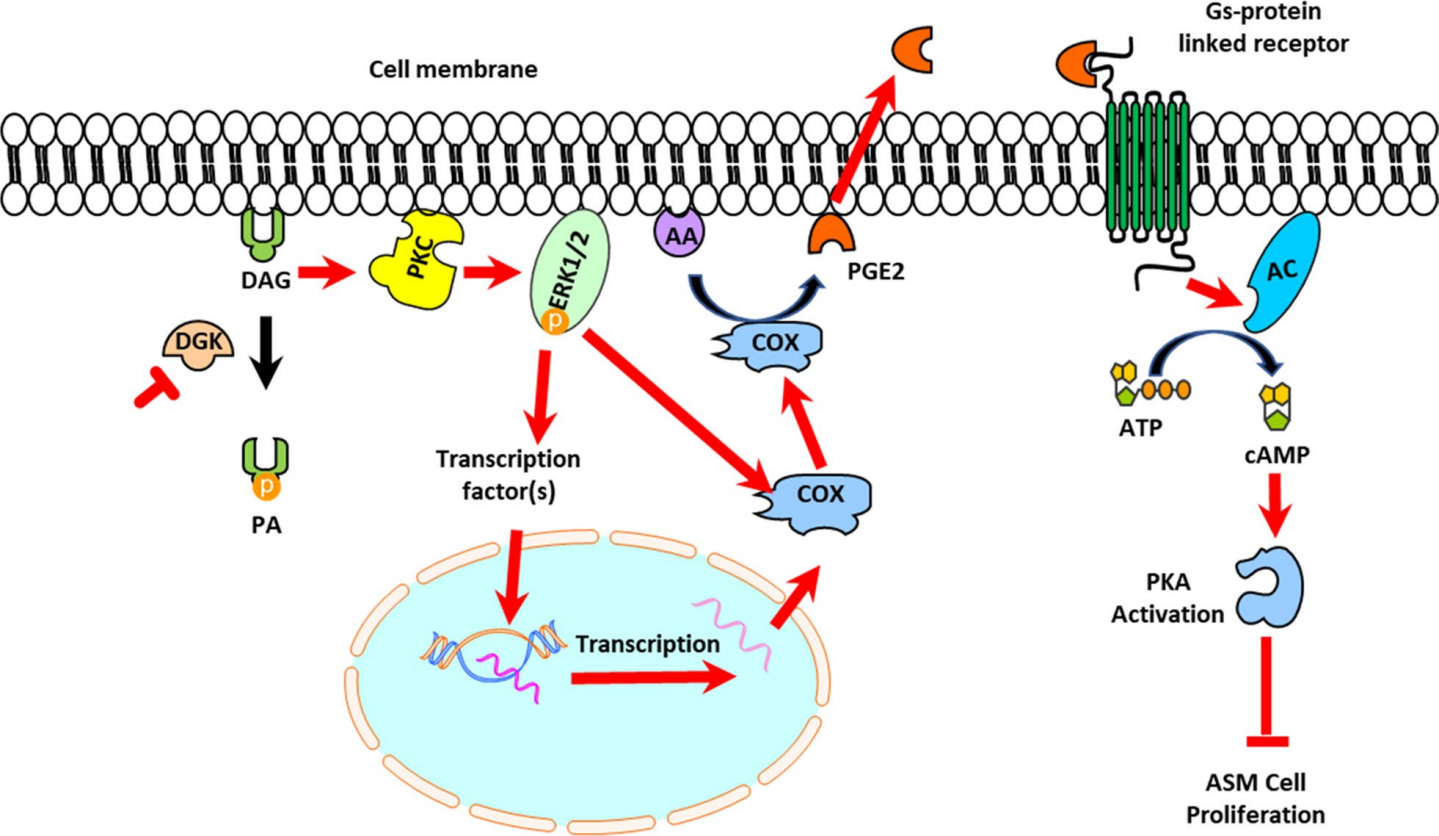
DGK inhibition mitigates airway smooth muscle cell proliferation by inducing anti-mitogenic signaling involving PKA. The proposed mechanism demonstrates that enhanced accumulation of DAG levels by DGK inhibitor induces PGE_2_ secretion via a PKC-ERK1/2-COXII axis. Secreted PGE_2_ potentiates PKA-mediated anti-mitogenic signaling by activating Gs-coupled GPCRs

ASM cell proliferation is regulated by growth factors and GPCR agonists, and induction of protein kinase A (PKA) signaling mitigates growth-factor-mediated ASM cell growth and migration [22, 24, 38]. Gs-coupled GPCR agonists such as β-agonists and PGE_2_ are predominant inducers of PKA activity in ASM cells. Our studies have also shown that PGE_2_ is one of the strongest inducers of cAMP-PKA signaling in ASM cells, potentially explaining its superior anti-mitogenic effect [24]. We previously reported DGK inhibition attenuates ASM cell proliferation by inhibiting mitogenic signaling pathways induced by second-messenger phosphatidic acid [19]. In the present study, we sought to delineate the anti-mitogenic mechanisms of DGK inhibition in ASM cells. We demonstrate that treatment of ASM cells with DGK inhibitor I induces expression of COXII which results in increased production of PGE_2_. We further demonstrate that COXII expression is mediated by increased PKC and ERK1/2 activity, associated with increased DAG levels. We propose that DGK inhibition increases DAG membrane phospholipid levels, which in turn activates PKC/ERK1/2-mediated induction of COXII expression and production of PGE_2_. PGE_2_ activates Gs-coupled GPCR signaling and induces PKA activity, attenuating ASM cell proliferation (Fig. 5). These findings are consistent with our previous study showing DGK inhibition reduces ASM cell contraction by COXII-mediated PGE_2_ production and activation of Gs-coupled GPCR signaling [18]. DGK-mediated regulation of ASM contraction via COXII induction was observed when the cells were treated with DGK inhibitor for 15 min. In this study, we demonstrate that COXII induction by DGK inhibition persists for an extended period to influence ASM cell proliferation.

ASM cell pro-mitogenic signaling is canonically mediated by growth factor-activated ERK1/2 and PI3K signaling, and Gq signaling can cooperate with RTK signaling to synergistically promote ASM cell proliferation [4, 5, 13]. In terms of second messengers, membrane phospholipids including DAG are generated upon stimulation of ASM cells with extracellular stimuli and play a role in Gq and RTK signal transduction in ASM cells. DAG species are generated by activation of Gq-coupled GPCR and RTK signaling and are regulated by DGK, a lipid kinase [14, 39]. Inhibition of DGK increases DAG levels in ASM cells and is expected to further promote pro-mitogenic signaling. In contrast, our present and recently published studies suggest that DGK inhibition attenuates ASM cell proliferation [19]. Gs-coupled GPCR signaling via activation of PKA has been shown to inhibit mitogenic signaling in ASM cells [22–24]. Therefore, in this study, we explored the potential role of PKA in the anti-mitogenic effect of DGK inhibition.

We employed a well-established genetic approach to inhibit PKA activity in ASM cells involving a stable expression of PKA inhibitory peptide, PKI [23]. Our data suggest that DGK-mediated regulation of ASM cell proliferation indeed involve PKA activation. However, PKA activation in ASM cells is mediated by Gs-coupled GPCRs that activate PKA by increasing cAMP levels and regulate cell proliferation by modulating several downstream signals [22, 23]. The mechanism by which PKA is activated when DGK is inhibited was the focus of the subsequent studies. Our findings demonstrate that DGK inhibition promotes COXII-mediated production of PGE_2_ which in turn activates Gs-PKA signaling and inhibition of proliferation of ASM cells in addition to what we have shown in our published paper wherein we reported a COXII induction and PKA activation in the context of regulation of ASM contraction by acute inhibition of DGK.

We further explored the mechanism by which DGK inhibition results in COXII induction. First, DGK inhibition results in upregulation of COXII protein for 24 h suggesting a prolonged activation. Previously, we showed that acute inhibition of DGK induces COXII-PGE_2_-PKA-mediated relaxation of ASM cells [17]. Sustained induction of COXII by DGK inhibition are relevant in the context of cell proliferation, secretion, and migration, all of which are important in airway diseases. Next, we investigated the mechanism by which COXII is activated by DAG/DGK. In this context, our studies suggested a role for PKC and ERK1/2 in activating PKA signaling in ASM cells when the cells are treated with DGK inhibitor. In the presence of DGK inhibitor alone we demonstrate increased production of COXII which was significantly decreased in the presence of either PKC or MEK/ERK inhibitors. PKC containing a DAG-binding C1 domain is a major target protein of DAG [40, 41] and increased DAG levels in cells in the presence of DGK inhibitor is expected to enhance PKC activity. Further, previous studies show a crosstalk between PKC and ERK1/2 downstream of DAG [33]. Enhanced DAG levels in cells presumably augmented PKC and ERK1/2 activity when cells were treated with DGK inhibitor. Activation of PKC/ERK1/2 has been shown to play an important role in the induction of COXII expression, a mechanism by which prostaglandins are produced in the cell [37]. Our findings presented herein are consistent with the previously published literature implicating the role of PKC/ERK1/2 in COXII induction. In fact, we demonstrate that DGK inhibition transiently activate ERK1/2 in ASM cells. Similarly, in ASM cells, activation of ERK1/2 MAPK signaling was shown to be necessary for cytokine-induced COXII expression and PGE_2_ production [36, 42, 43].

In the present studies, we observed an increase in COXII expression and PKA activation in the presence of a DGK inhibitor, all of which were decreased in the presence of PKC and MEK/ERK1/2 inhibitors. This further highlights the potential cross-talk between PKC and ERK1/2 in regulating cell function [33], however, it also raises the question of compartmentalized signaling complexes. While PKC proteins are translocated from cytosol to membrane upon activation, ERK1/2 signaling remains cytosolic. PKC activation is likely upstream in the context of DGK inhibition considering PKC has a DAG-binding domain and move closer to membrane upon stimulation of cells by an external signal. Additionally, DGK enzyme activity is regulated by (i) second messengers (Ca^2+^) (ii) protein-protein interactions (PKC MARCKS domain) or (iii) cellular sub-localization [39, 44, 45]. It is probable that the proximity of DGK and PKC to the cell membrane allow for feed-forward signaling inducing a stronger, more vigorous signal inducing COXII production. Future studies characterizing the compartmentalization of these proteins are necessary to confidently draw conclusions.

While our studies demonstrated induction of COXII expression under DGK inhibition, we did not ascertain the other mechanism by which COXII activity can be regulated. This includes posttranslational modifications such as phosphorylation, glycosylation, nitrosylation, intracellular trafficking, ubiquitination, and ER-associated degradation (ERAD) [32, 46]. Studies have reported phosphorylation events of COXII increase PGE_2_ production [34], and others suggested that protein-protein interactions modulate COXII activity without change in COX protein expression [47]. Interestingly, studies highlighted an important role for DGKε isoform in kidney, brain and adipose tissue to induce COXII expression and activity [48–50]. Although isotype specific role of DGK in this study was not assessed, our study suggest that DGK inhibition promotes PKA activation and inhibition of proliferation of ASM cells. Both DGK α and ζ isoforms could potentially contribute to ASM cell proliferation.

## Conclusions

Overall, the present studies allude to a novel anti-mitogenic mechanism for ASM cells in the presence of a DGK inhibitor mediated by PKA activation involving PKC/ERK1/2-mediated induction of COXII and PGE_2_ secretion. This leads to the paracrine/autocrine activation of Gs-coupled GPCRs, presumably EP-2 and -4 receptors, resulting in inhibition of ASM cell proliferation by PKA. Our current and previously published studies further advance DGK as a potential target for mitigating ASM-mediated pathophysiological responses such as airway remodeling and hyperresponsiveness in airway diseases, including asthma.

## Data Availability

All data in the manuscript is available through the responsible corresponding author.

## Abbreviations

DGK: Diacylglycerol kinase
DAG: Diacylglycerol
ASM: Airway smooth muscle
PKA: Protein kinase A
DGK I: Diacylglycerol kinase inhibitor I
PGE_2_: Prostaglandin E_2_
GFP: Green fluorescent protein
PKI: PKA inhibitory peptide
PDGF: Platelet-derived growth factor
COXII: Cyclooxygenase 2
VASP: Vasodilator-stimulated phosphoprotein
CREB: cAMP-response element binding protein
PKC: Protein kinase C
ERK: Extracellular signal-regulated kinase
GPCR: G protein-coupled receptor
RTK: Receptor tyrosine kinase
PA: Phosphatidic acid
ATP: Adenosine triphosphate
cAMP: Cyclic adenosine monophosphate
VSV-G: p-Vesicular stomatitis virus-G vector
EP2, -4: Prostaglandin EP2, EP4 receptors
PI3K: Phosphoinositide 3-kinase
